# Explosive breeding in tropical anurans: environmental triggers, community composition and acoustic structure

**DOI:** 10.1186/s12898-019-0243-y

**Published:** 2019-07-19

**Authors:** Juan Sebastian Ulloa, Thierry Aubin, Diego Llusia, Élodie A. Courtois, Antoine Fouquet, Philippe Gaucher, Sandrine Pavoine, Jérôme Sueur

**Affiliations:** 1 Institut Systématique Evolution Biodiversité (ISYEB), Muséum National d’Histoire Naturelle, CNRS, Sorbonne Université, EPHE, 57 Rue Cuvier, CP 50, 75005 Paris, France; 2 Equipe Communications Acoustiques, UMR 9197, Neuro-PSI-CNRS, Université Paris-Sud, Bat.446, 91405 Orsay, France; 3 Terrestrial Ecology Group (TEG), Departamento de Ecología, Facultad de Ciencias, Universidad Autónoma de Madrid, C / Darwin, 2, Edificio de Biología, C-211, Ciudad Universitaria de Cantoblanco, 28049 Madrid, Spain; 4 Laboratoire Ecologie, Évolution et Interactions des Systèmes Amazoniens (LEEISA) UMSR 3456 (CNRS/IFREMER/Université de Guyane), Centre de recherche de Montabo, Université de Guyane, 275, route de Montabo, Cayenne, BP 70620, 97334 CAYENNE Cedex France; 5 Laboratoire Evolution & Diversité Biologique, Université Toulouse III Paul Sabatier, Bâtiment 4R1, 118, Route de Narbonne, 31062 Toulouse Cedex 9, France; 6 Centre d’Ecologie et des Sciences de la Conservation (CESCO), Muséum National d’Histoire Naturelle, CNRS, Sorbonne Université, 57 Rue Cuvier, CP 135, 75005 Paris, France

**Keywords:** Acoustic diversity, Anuran community, Ecoacoustics, Biodiversity monitoring

## Abstract

**Background:**

Anurans largely rely on acoustic communication for sexual selection and reproduction. While multiple studies have focused on the calling activity patterns of prolonged breeding assemblages, species that concentrate their reproduction in short-time windows, explosive breeders, are still largely unknown, probably because of their ephemeral nature. In tropical regions, multiple species of explosive breeders may simultaneously aggregate leading to massive, mixed and dynamic choruses. To understand the environmental triggers, the phenology and composition of these choruses, we collected acoustic and environmental data at five ponds in French Guiana during a rainy season, assessing acoustic communities before and during explosive breeding events.

**Results:**

We detected in each pond two explosive breeding events, lasting between 24 and 70 h. The rainfall during the previous 48 h was the most important factor predicting the emergence of these events. During explosive breeding events, we identified a temporal factor that clearly distinguished pre- and mid-explosive communities. A common pool of explosive breeders co-occurred in most of the events, namely *Chiasmocleis shudikarensis*, *Trachycephalus coriaceus* and *Ceratophrys cornuta*. Nevertheless, the species composition was remarkably variable between ponds and for each pond between the first and the second events. The acoustic structure of explosive breeding communities had outlying levels of amplitude and unexpected low acoustic diversity, significantly lower than the communities preceding explosive breeding events.

**Conclusions:**

Explosive breeding communities were tightly linked with specific rainfall patterns. With climate change increasing rainfall variability in tropical regions, such communities may experience significant shifts in their timing, distribution and composition. In structurally similar habitats, located in the same region without obvious barriers, our results highlight the variation in composition across explosive breeding events. The characteristic acoustic structure of explosive breeding events stands out from the circadian acoustic environment being easily detected at long distance, probably reflecting behavioural singularities and conveying heterospecific information announcing the availability of short-lived breeding sites. Our data provides a baseline against which future changes, possibly linked to climate change, can be measured, contributing to a better understanding on the causes, patterns and consequences of these unique assemblages.

## Background

Amphibians are currently the most endangered group of vertebrates, with more than 32% of species classified as at risk of extinction [1–3]. Recent investigations on the causes of amphibian declines have identified the role of climate change on a global scale [4–7]. In addition to the climate-linked epidemic hypothesis, research has focused on the effect of climate change on behaviour, reproduction and distribution of amphibians [8, 9]. As ectotherms, alterations on temperature and rainfall regimes might strongly affect key aspects of amphibian life cycles, even jeopardizing their survival [10]. Both theoretical and experimental studies suggest that low latitude ectothermic species are more vulnerable to climate changes than their higher latitude counterparts [11]. Tropical species indeed tend to have narrower thermal tolerance [12] and their actual habitat temperatures are closer to their upper thermal limit [10, 13]. Even slight changes in environmental conditions might therefore have a strong effect on these tropical species [14].

Anurans largely rely on acoustic communication for sexual selection and reproduction [15, 16]. Studies have revealed that temporal patterns of calling and breeding activity of anurans are influenced by multiple environmental factors, such as temperature, humidity or light intensity [17–19]. Moreover, recent findings have also shown that photoperiod might be an important driver of the calling activity of numerous anuran species [20–23]. The response to abiotic environmental factors may vary between species and according to the reproduction strategy [18, 24]. While some anurans show long periods of calling activity and mating, known as prolonged breeders, others concentrate their reproduction during short time windows, even a few hours per year, and are known as explosive breeders [24]. In tropical regions, massive aggregations of explosive breeders generally involve multiple species simultaneously, leading to highly-diverse anuran communities [25–28]. Such phenomena typically occur in ephemeral ponds, which are sparsely distributed in tropical forests and are likely triggered under particular weather conditions.

Yet, the structure and dynamics of these unique acoustic communities are still largely unknown probably because of their ephemeral nature, density and complexity. To our best knowledge, few studies have documented broad and generic patterns in explosive neotropical anurans, observing correlations between peaks of activity and the occurrence of heavy rainfall at the beginning of the rainy season [25, 26, 28], and only two studies have analysed their fine scale dynamics [23, 27]. In the former study, the data collection was done by human calling surveys through a 4-month fieldwork in French Guiana. Gottsberger and Gruber in 2004 identified temporal partitioning within the anuran community according to their reproductive modes [27]. In particular, the group of species with aquatic oviposition presented sporadic acoustic activity following heavy rainfall, a phenomenon that occurred twice during the study. But their study focused on two close-by ponds, less than 240 m apart, limiting the interpretation of the results. Replications at spatial and temporal dimensions are crucial to examine the constitution and diversity of these communities, to decipher their dynamics and to identify their link with environmental factors. Schalk and Saenz in 2016 examined the calling phenology of anurans in the Gran Chaco ecoregion at seven ponds during 9 months with passive acoustic sensors. For explosive breeding species they found that calling activity was positively and significantly correlated with at least two abiotic factors, rainfall and photoperiod [23]. Calling individuals gathering around breeding points form dense choruses characterized by a complex acoustic structure, broad masking interference and high sound pressure level [29]. Choruses formed by tropical anurans in explosive breeding events are extreme on these features due to the extraordinary species diversity and density of calling males [27]. Such assemblages constitute unique examples of multi-species choruses presumably eliciting complex interspecific interactions.

The technical difficulty in monitoring simultaneously these ephemeral communities has been one of the reasons for the lack of a wider geographic coverage. Traditional field-based observations are not scalable, thus it is crucial to adapt and test cost-effective methods. More than 20 years ago the idea of using automated data acquisition methods to monitor amphibians was already proposed [30], but it is only recently, thanks to the development of reliable passive acoustic sensors that this method has gain popularity [31–36]. These acoustic sensors can be programmed to record for days or even months in a non-invasive and cost-efficient way, so that replication in time and space is now possible. Most anuran amphibians produce loud, stereotyped, and species-specific advertisement calls for mate attraction. These acoustic signals can be therefore remotely recorded to monitor populations as testified by several studies on temperate (e.g. [8, 18, 37]) and tropical species (e.g. [17, 38–40]).

Using automated sensors, we collected for the first time acoustic and environmental data to monitor simultaneously and regularly explosive breeding events in tropical anuran communities, at five temporary ponds located along the Kaw Mountain in French Guiana. This systematic passive acoustic monitoring allowed us to tackle key ecological questions related to the patterns, causes and consequences of such a striking phenomenon. We specifically addressed four questions: (1) What are the main meteorological factors that trigger the emergence of explosive breeders? (2) Which species co-occur before and during explosive breeding events? (3) What is the variation in the acoustic community composition within and between sites? (4) What are the main acoustic patterns, spectral characteristics and diversity before and during explosive breeding events? Answering these questions may shed light on the potential selective pressures shaping these complex acoustic communities.

## Methods

### Study site

We monitored explosive breeding assemblages in the lowland tropical rainforest of French Guiana, along the Kaw Mountain (4°36′N; 52°16′W). As in most regions located close to the equator, seasonal climatic variations in the study site were primarily due to changes in rainfall and humidity. The climate regime is characterized by two periods of rainfall: the main rainy season takes place from mid-November to the end of February and a less marked rainy season occurs from April to July. For this study, we collected acoustic and environmental data from the end of the dry season (10 November 2015) to the end of the main rainy season (16 February 2016).

**Fig. 1:**
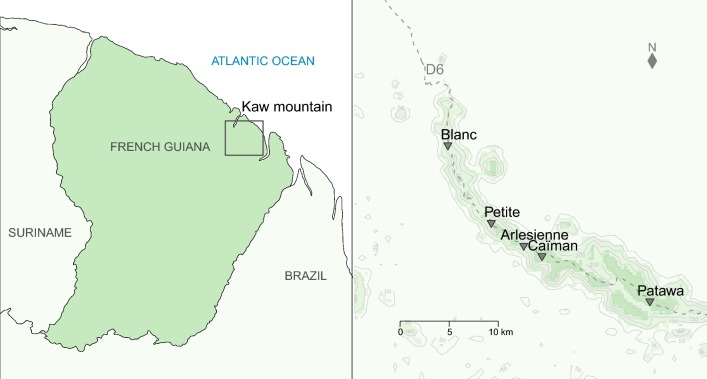
Location of the study area. On the left, location of the Kaw mountain in French Guiana. On the right, location of the five study sites along a 30.4 km transect next to the departmental road D6. GIS shape files were obtained from the National Institute of Geographic and Forestry Information (http://professionnels.ign.fr/)

We focused the sampling on five seasonal ponds along a 30.4 km transect corresponding to the departmental road D6 (Fig. 1). These temporary shallow water bodies are flooded during the rainy seasons and then dry out predictably during periods of low rainfall, July to November. The ponds were surrounded by dense tropical forest, located between 236 and 313 m above the sea level, and had distinct sizes, from 224.8 to 2240.2 m^2^ (Table 1).

**Table 1:** Altitude, location, and area of the five study ponds

Local name	Code name	Altitude	GPS coordinates	Area
Caïman	Ca	313	4°34′10″N; 52°13′11″W	1192.3
Blanc	Bl	236	4°40′14″N; 52°18′22″W	399.5
Patawa	Pa	295	4°31′41″N; 52°07′14″W	2240.2
Arlesienne	Ar	269	4°32′44″N; 52°14′11″W	672.0
Petite	Pe	289	4°35′59″N; 52°15′59 W	224.8

Altitude is given in meters above sea level (m a.s.l.) and area in m^2^

### Sampling protocol

We monitored anuran calling activity and weather conditions simultaneously in each pond using automated sensors with a regular sampling schedule. To record the acoustic communities, we placed on the edge of each pond at breast height an automated sound recorder equipped with an omnidirectional microphone (SM2, Wildlife Acoustics, Inc., Concord, MA, USA). The device was set up to record data 1 min every 29 min, at 44.1 kHz and 16 bit resolution, so that we obtained 5616 recordings for each pond.

To register local abiotic environmental data, we placed next to the sound recorder a data logger (H21-002, Onset) equipped with sensors to measure three weather variables: rainfall (Onset, S-RGB-M002), temperature, and relative humidity (Onset, S-THB-M008). In addition, we retrieved two global environmental variables, atmospheric pressure (PTB220, Vaisala) and solar radiation (CMP6, Kipp and Zonen), from the nearest weather station at the Félix Eboué airport (4°50′N; 52°22′W), 19 km from the study site.

### Time-series analysis

Because of the emergence of a great number of males from multiple amphibian species, explosive breeding events are known to produce a remarkably loud chorus. Therefore, we identified the occurrence of explosive breeding events in the audio recordings by searching for outlier amplitude peaks. The overall amplitude of each recording was measured by computing the root-mean-square of the signal amplitude envelope. Then, we applied a median filter with a 24-h window and we searched for outliers in the resulting smoothed time series. The outliers were defined as values distributed one-and-a-half times the inter-quartile range (IQR) above the third quartile (Q3 + 1.5 × IQR). Every outlier event was inspected by listening to the recordings to confirm the presence of an explosive breeding event.

Preliminary analyses showed clear and steep increase on the sound pressure level during explosive breeding events resulting from the increase in calling activity from anuran communities. While the beginning of the explosive breeding events exhibited constant and exceptionally high call rate for around 24 h, calling activity later presented multiple oscillations before ceasing or returning to common levels. In order to have comparable sections for each event and compare pre- and mid-explosive breeding communities, we focused our subsequent analysis on a 48 h window, starting 24 h before the onset and ending 24 h after the onset of explosive breeding events.

We used a machine-learning framework to test whether the occurrence of the explosive breeding events could be predicted by abiotic factors. Weather conditions were considered as predictor variables and the triggering dates of the explosive breeding events as a binary response variable. The abiotic variables comprised low-level and high-level features. Low-level features were the raw quantitative meteorological measurements from the on-site sensors and the weather station, namely temperature, temperature variation, relative humidity, rainfall, atmospheric pressure, atmospheric pressure variation, photoperiod and solar radiation. Since the emergence of the breeding events can also be due to previous environmental conditions, we also included high-level features in the statistical analyses calculated based on the raw climatic data. These high-level features were the lagged-variables, previous 24, 48, and 72 h, and past-cumulative variables from the previous 48 and 72 h. The final predictor matrix included 48 variables with 466 observations. We measured prediction accuracy and variable importance on classification using the random forest statistical classifier [41]. We assessed the importance of the predictor variables by comparing the difference in misclassification error (mean decrease accuracy) between the original data and a permuted set of data. The modified data for each predictor variable consisted in randomly permuted observations that are passed down the random forest. The higher the decrease in accuracy between the original and the modified data, the higher the importance of the predictor variable [42].

### Community diversity analysis

We investigated temporal and spatial variation on the diversity and composition of the acoustic communities of explosive breeding events. We define a community as the set of species heard at a given time interval on a given pond. For each event, we systematically discretized the temporal gradient of 48 h into four temporal periods of 12 h. A first period (t1) ranged from 24 to 12 h before the explosive breeding event, a second period (t2) ranged from 12 h before to the onset of the event, a third period (t3) enclosed the first 12 h of the event, and a fourth period (t4) ranged from 12 to 24 h after the onset of the event.

We then sub-sampled our database by choosing one recording every 2 h, for a total of 240 recordings of 60 s. Three of us (EC, AF and PG), who are highly trained in aural identification of anuran species of French Guiana, scrutinized each recording and annotated the occurrence of calling species. A final presence-absence vector was derived for each recording by majority voting, thereby, potential observer bias was prevented while the accuracy of the annotations enhanced. This phase led to the identification of a total of 25 species.

We used the crossed-DPCoA [43], an ordination method that provides an approach for analysing the effects of crossed factors on the diversity of communities, to identify the effects of external factors on community composition. Here we analysed the effect on the species composition of amphibian communities of the time period before or after the event (t1, t2, t3, t4), and the event (an event is one of the two breeding explosions observed at a given pond). The time period and the event are two crossed factors. The aim of crossed-DPCoA is to visualize the pattern of diversity due to a factor A knowing the existence of a crossed factor B. DPCoA helps to visualize the main effect of factor A, here species composition, and the effect of the interaction between A and B, removing the main effect of factor B. The method first defines a space where species, communities and the levels of the two factors are visualized as points. Then, the communities are positioned at the centroid of their constitutive species, and the levels of the factors at the centroid of communities associated with them. The method then searches for principal axes of the levels of factor A, retaining potential effects of the interaction between A and B, but removing the main effect of factor B. In particular, we used the first version of DPCoA, which eliminates the effect of factor B by moving this factor to the centre of the space. We analysed first the effect of the events on the species composition of amphibian communities given the time period and then the effect of the time periods given the event.

### Acoustic diversity analysis

To further compare the anuran acoustic assemblages of the pre- and mid-explosive breeding events, we followed the same previous procedure while adding information related to the acoustic dissimilarities between species. We used the same community data and repeated the ordination analysis. However, here we did not consider species as equidistant in the space of the crossed-DPCoA, we used the acoustic properties of the calls of the species to define acoustic dissimilarities between pairs of species. In this defined space, the distance between two species-specific points is a measure of the acoustic dissimilarity.

We estimated the acoustic dissimilarity between two species using focal recordings of each species-specific call available from personal field recordings (PG, EC, AF, JSU; n = 17) and from commercial recordings ([44], n = 8). We selected recordings that met two criteria: (1) the call had to be emitted by an isolated individual, and (2) the signal-to-noise-ratio (SNR) of the signal had to be higher than 30, where SNR = 20 log_10_(RMS_signal_/RMS_noise_) and RMS is the root-mean-square amplitude of the signal. Then, the spectral composition of each call was quantified by computing a short-time Fourier transform (FFT length of 512, no overlap, Hanning window), averaging the columns of the subsequent matrix (the temporal dimension), and applying a log-transformation. The acoustic dissimilarity between the species call was assessed by computing the cumulative dissimilarity of the spectral distributions or index D_cf_ [45].

In addition, we analyzed the spectral profiles of the recordings collected in the field to investigate the changes in the acoustic environment before and during explosive breeding events. We first calculated the mean spectrum of each file. Then, we compared the spectral profiles at different moments of the explosive breeding event using a random forest procedure. We quantified and evaluated the classification accuracy and the importance of each feature, here each spectral profile, for the classification using the random forest importance measure [42].

Finally, we estimated the α diversity of each acoustic community by computing the species richness, the Gini–Simpson coefficient, and the quadratic entropy. The richness is the number of species in the community. The Gini–Simpson index takes into account the number of species and their proportions [46, 47]. The quadratic entropy, or Rao’s diversity coefficient [48], is based on the number of species, their proportions and incorporates a between-species dissimilarity matrix (here the pair-wise acoustic dissimilarities). For each diversity index, we tested the differences among periods of the explosive breeding event (i.e. t1, t2, t3 and t4) and between events (i.e. the first and second event per site), as well as the interaction between both factors, with repeated-measures ANOVA. Shapiro–Wilk and Mauchly tests revealed no violation of the assumptions of normality and sphericity, respectively, when using ANOVA tests (in all cases: W > 0.76, df = 5, p > 0.05; *X*^*2*^ < 0.02, df = 5, p > 0.05). Tukey test with Bonferroni correction was finally performed as post hoc procedure to examine pairwise comparisons between time periods. The type I error was set at a nominal level of 5%.

Acoustic and statistical analyses were computed using the R software [49]. In particular, spectral audio features and dissimilarity matrices were computed using the seewave R-package [50], community and diversity ordination analyses were calculated with the adiv R-package [51], and statistical classification was computed with the random forest R-package [52].

## Results

### Time series analysis

**Fig. 2:**
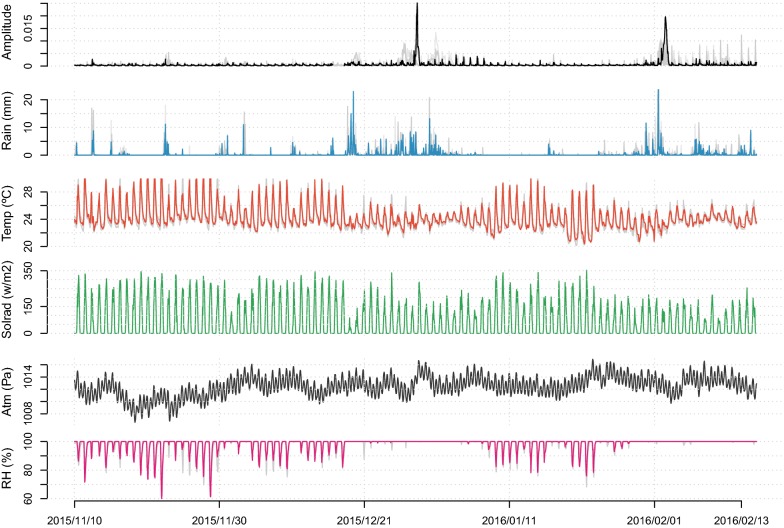
Temporal fluctuation of the measured environmental variables: sound pressure amplitude (root mean square of the signal amplitude envelope), rainfall (mm), temperature (°C), solar radiation (w/m^2^), atmospheric pressure (Pa) and relative humidity (%). The acoustic amplitude plot shows two clear peaks that are related to explosive breeding events. For illustration purposes, only the variables measured at a single pond (Blanc) are highlighted in dark colours, the data collected at the other four ponds are plotted as light grey lines in the background. Dates are given as year/month/day

Sound pressure level showed regular 24-h cycles during the study (Fig. 2). Yet, this regularity was interrupted by abrupt and steep increases in the amplitude lasting between 24 and 70 h that occurred at the end of December 2015 and the beginning of February 2016. Rainfall was irregularly distributed during the study showing two major rainfall events, the first one between 19 December 2015 and 4 January 2016, and the second one from 23 January to 15 February 2016. During those periods, daily fluctuations in temperature were less pronounced, solar radiation was lower, and relative humidity remained close to 100% (Fig. 2).

**Fig. 3:**
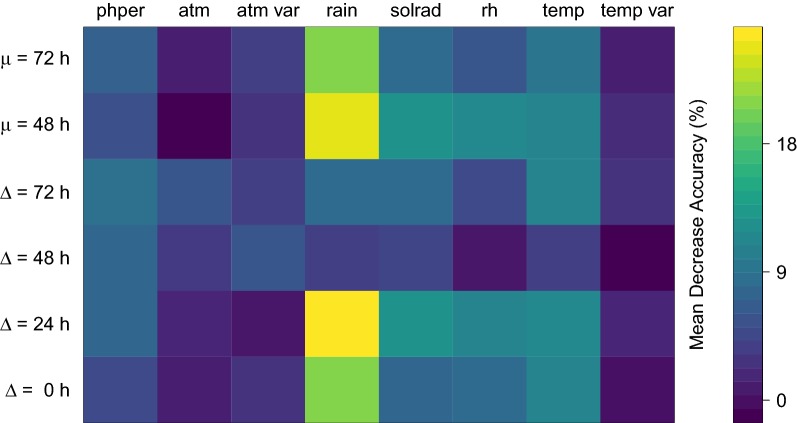
Variable importance measure (mean decrease accuracy) from random forest classification used for predicting the start of the explosive breeding events. Variables with higher values were more important for the classification. A total of 48 environmental variables were evaluated based on the combination of eight measurements and six derived variables. The variables measured were: photoperiod (phper), atmospheric pressure (atm), atmospheric pressure variance (atm var), rainfall (rain), solar radiation (solrad), relative humidity (rh), temperature (temp) and temperature variance (temp var). The derived variables were based on their delay (Δ) and persistence (μ) along the time (0, 24, 48 and 72 h)

Applying an amplitude filter, we detected in each pond two major explosive events, i.e. 10 in total, lasting between 24 and 70 h, later confirmed by aural evaluation. Using the combined meteorological variables (instant, lagged and past-cumulative) and the random forest classifier, we were able to accurately predict the emergence of all (100%) explosive breeding events with a low false positive rate of 9.6% for out-of-the-bag estimates, that is using observations that were not used to build the predictive model. Variable importance ranking showed that rainfall was the most influential weather determinant, in particular, the amount of rain during the previous 24 h and most importantly the past-cumulative rainfall during the previous 48 to 72 h (Fig. 3). The rest of the variables (temperature, relative humidity, atmospheric pressure, photoperiod and solar radiation) had minor predictive power.

### Community diversity analysis

**Fig. 4:**
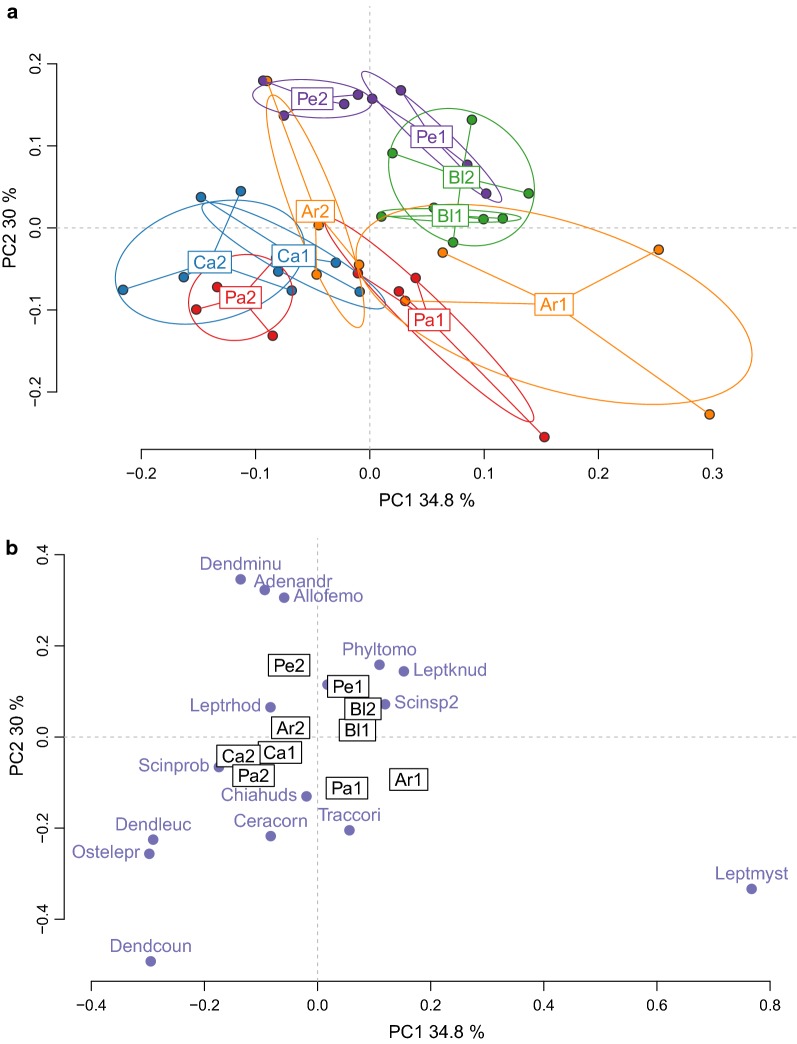
Diversity of the species composition in explosive breeding events across sites. The two principal axes (64.8% of variance explained) of the crossed DPCoA analysis are plotted. **a** Diversity between and within communities. Each point is a community. The communities were color-coded with the levels of the factor event. Code names for events are: Ar = Arlesienne, Bl = Blanc, Ca = Caïman, Pa = Patawa, Pe = Petite. The number that follows the code name distinguishes the explosive breeding event in each site, for instance Ar1 is for the first event on site Arlesienne, and Ar2 is for the second event on the same site. **b** Coordinates of the constitutive species in the axes. Each point is a species. Only the species that had the highest values on the axes were named. Code names for the species are: *Adenomera andreae* = Adenandr, *Allobates femoralis* = Allofemo, *Ceratophrys cornuta* = Ceracorn, *Chiasmocleis hudsoni* = Chiahuds, *Dendropsophus counani* = Dendcoun, *Dendropsophus leucophyllatus* = Dendleuc, *Dendropsophus minutus* = Dendminu, *Leptodactylus knudseni* = Leptknud, *Leptodactylus mystaceus* = Leptmyst, *Leptodactylus rhodomystax* = Leptrhod, *Osteocephalus leprieurii* = Ostelepr, *Phyllomedusa tomopterna* = Phyltomo, *Scinax sp2* = Scinsp2, *Trachycephalus coriaceus* = Traccori

We first analysed the species composition of explosive breeding events using crossed-DPCoA, which allowed to focus on the explosive breeding events removing the effect of the crossed factor linked to the time period before or after the event. The first two principal axes expressed respectively 34.8% and 30% of the main effect variability of the factor site (Fig. 4a). Neither the first nor the second axis presented a particular pattern, the explosive breeding events having largely overlapping communities. Nevertheless, some sites (Patawa, Arlesienne and Petite) presented high between-event diversity, each explosive breeding event having a particular and unique combination of species (Fig. 4b). Inter-site and intra-site variability of the explosive breeding events for these sites had the same order of magnitude.

**Fig. 5:**
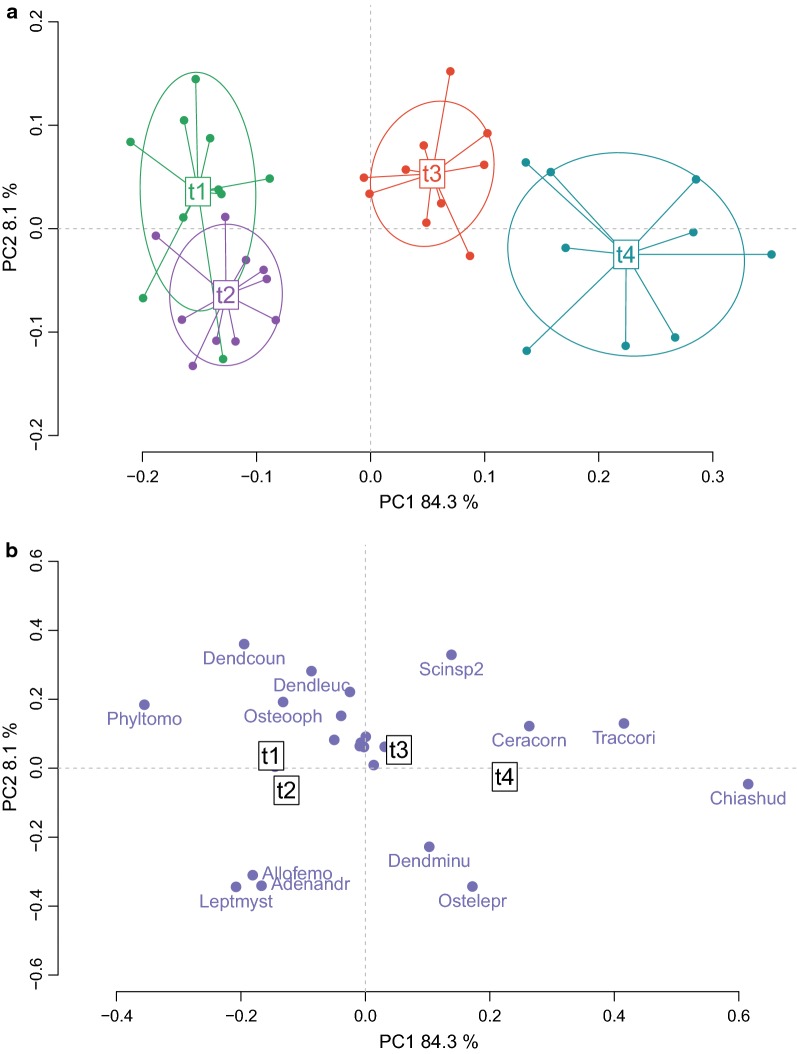
Diversity of the species composition in explosive breeding events across time. The two principal axes (92.4% of variance explained) of the crossed DPCoA analysis are plotted. **a** Diversity between and within communities along the time. Each point is a community. The communities are color-coded with the levels of factor time: t1, t2, t3, and t4. Pre-explosive and mid-explosive communities are clearly discriminated along the first axis. **b** Coordinates of the constitutive species in the axes. Each point is a species. Only the species that had the highest values on the axes were named: *Adenomera andreae* = Adenandr, *Allobates femoralis* = Allofemo, *Ceratophrys cornuta* = Ceracorn, *Chiasmocleis shudikarensis* = Chiashud, *Dendropsophus counani* = Dendcoun, *Dendropsophus leucophyllatus* = Dendleuc, *Dendropsophus minutus* = Dendminu, *Leptodactylus mystaceus* = Leptmyst, *Osteocephalus leprieurii* = Ostelepr, *Osteocephalus oophagus* = Osteooph, *Phyllomedusa tomopterna* = Phyltomo, *Scinax sp2* = Scinsp2, *Trachycephalus coriaceus* = Traccori

Then, to reveal the temporal variability in the communities, we eliminated the crossed effect of factor ‘event’ with the DPCoA. The calling activity of the anuran communities was structured along the temporal dimension (Fig. 5a). The first axis of the DPCoA, with 84.3% of variance explained, clearly discriminated two assemblages: the pre-explosive community (t1 and t2 on the negative side) and a characteristic explosive breeding community (t4 on the positive side). A transitional community with species from both sides appeared near the origin (t3). While the pre-explosive communities (t1 and t2) were partly similar in their species composition, t3 and t4 had clear and unique species composition. The species that characterized the pre-explosive community (t1 and t2) were *Phyllomedusa tomopterna*, *Leptodactylus mystaceus*, and *Dendropsophus counani* (Fig. 5b). Because they had positive coordinates on the first axis, the species that characterized the explosive breeding community (t4) were *Chiasmocleis shudikarensis*, *Trachycephalus coriaceus* and *Ceratophrys cornuta* (Fig. 5b). The transitional community (t3) showed an intermediate place on the ordination; these communities had a balanced mixed of pre-explosive and explosive breeding species.

### Acoustic diversity analysis

**Fig. 6:**
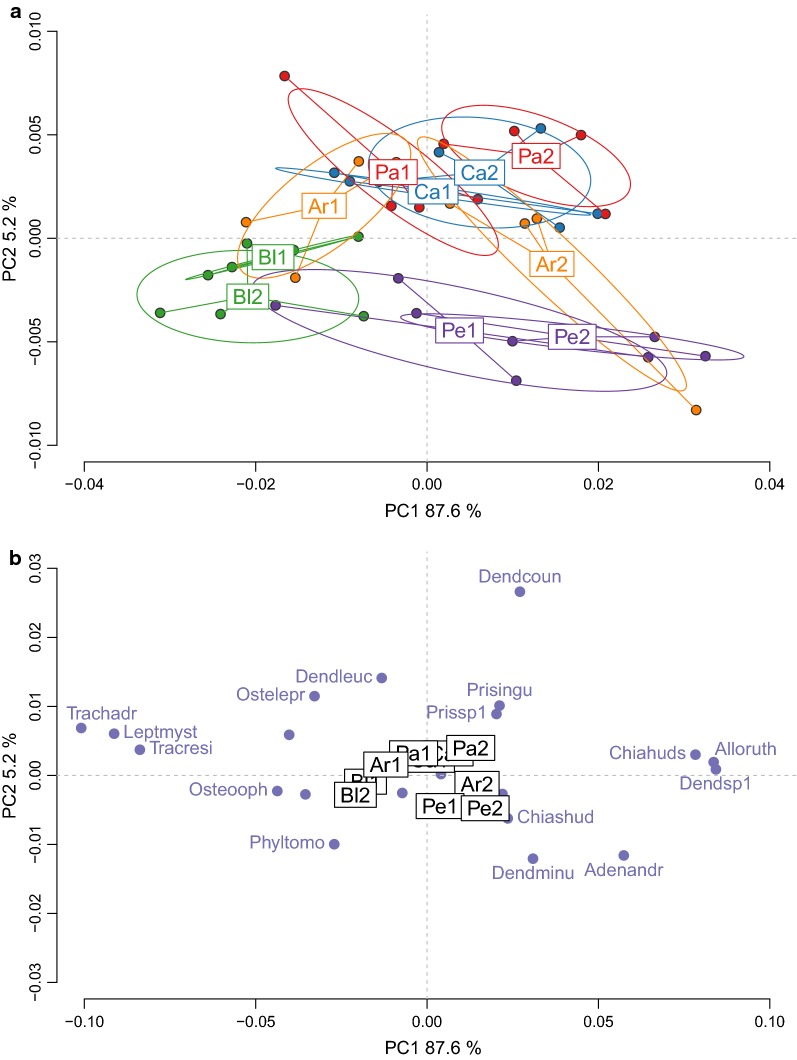
Diversity of the acoustic composition in explosive breeding events across sites. The two principal axes (92.8% of variance explained) of the crossed DPCoA analysis are plotted. **a** Diversity between and within communities. Each point is a community. The communities were color-coded with the levels of the factor event. Code names for events are: Ar = Arlesienne, Bl = Blanc, Ca = Caïman, Pa = Patawa, Pe = Petite. The number that follows the code name distinguishes the explosive breeding event in each site, for instance Ar1 is for the first event on site Arlesienne, and Ar2 is for the second event on the same site. **b** Coordinates of the constitutive species in the axes. Each point is a species. Only the species that had the highest values on the axes were named. Code names for the species are: *Adenomera andreae* = Adenandr, *Allophryne ruthveni* = Alloruth, *Chiasmocleis hudsoni* = Chiahuds, *Chiasmocleis shudikarensis* = Chiashud, *Dendropsophus counani* = Dendcoun, *Dendropsophus leucophyllatus* = Dendleuc, *Dendropsophus minutus* = Dendminu, *Dendropsophus sp1* = Dendsp1, *Leptodactylus mystaceus* = Leptmyst, *Osteocephalus leprieurii* = Ostelepr, *Osteocephalus oophagus* = Osteooph, *Phyllomedusa tomopterna* = Phyltomo, *Pristimantis inguinalis* = Prisingu, *Pristimantis sp1* = Prissp1, *Trachycephalus coriaceus* = Traccori, *Trachycephalus hadroceps* = Trachadr, *Trachycephalus resinifictrix* = Tracresi

As in the previous community analysis, we initially removed the effect of the crossed factor time. The first principal axis, with 87.6% of variance explained, was strongly correlated with the peak frequency of the calls (r = 0.96, Pearson correlation; Fig. 6). The crossed-DPCoA ordered the species with low frequency sounds on the left of the axis and species with high-pitched calls on the right. Distributed in this new space, the sites presented largely overlapping acoustic communities with a balance between high and low frequencies. Yet, the ponds Patawa, and Arlesienne had a high between-event acoustic diversity (Fig. 6a). At both ponds, the first explosive breeding event was characterized with lower frequencies than the second one.

**Fig. 7:**
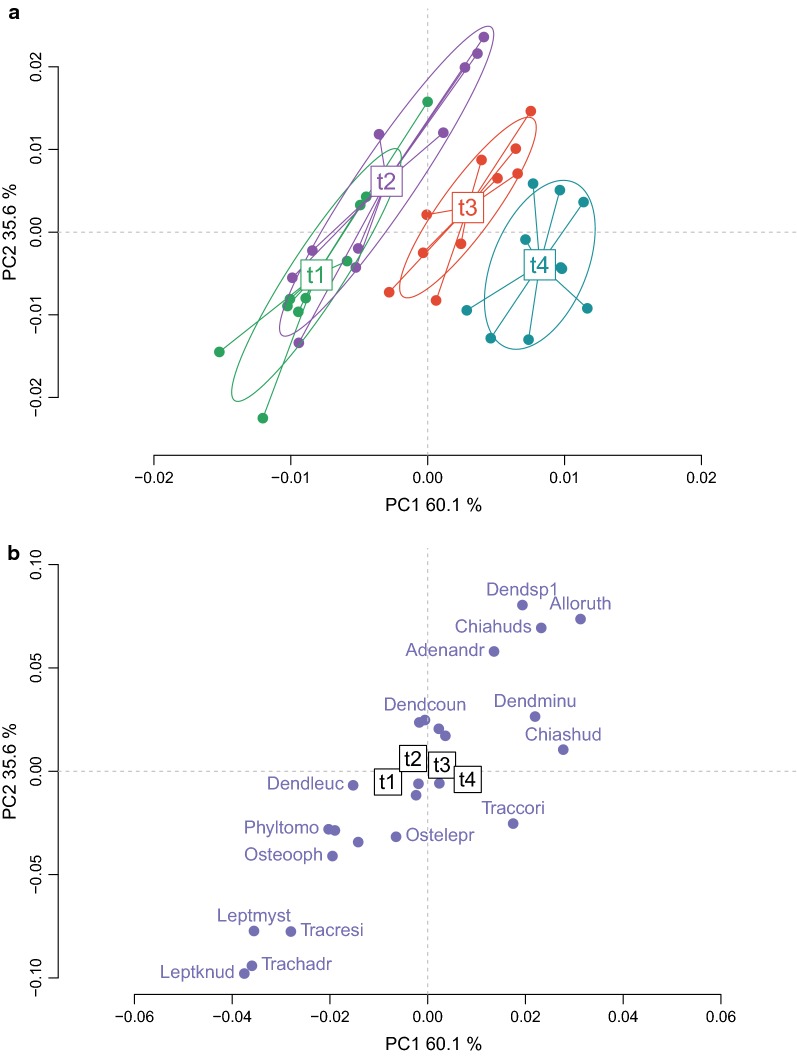
Diversity of the acoustic composition in explosive breeding events across time. The two principal axes (95.7% of variance explained) of the crossed DPCoA analysis were plotted. **a** Diversity between and within communities. Each point is a community. Communities were color-coded with levels of factor time: t1, t2, t3, t4. Time periods t1 and t2 range 24–12 h and 12–0 h respectively before the onset of explosive breeding events, t3 and t4 range 0–12 h and 12–24 h respectively after the onset. **b** Coordinates of the constitutive species-specific calls in the principal axes. Each point is a species. Only the calls with higher values on the axes were named: *Adenomera andreae* = Adenandr, *Allophryne ruthveni* = Alloruth, *Chiasmocleis hudsoni* = Chiahuds, *Chiasmocleis shudikarensis* = Chiashud, *Dendropsophus counani* = Dendcoun, *Dendropsophus leucophyllatus* = Dendleuc, *Dendropsophus minutus* = Dendminu, *Dendropsophus sp1* = Dendsp1, *Leptodactylus knudseni* = Leptknud, *Leptodactylus mystaceus* = Leptmyst, *Osteocephalus leprieurii* = Ostelepr, *Osteocephalus oophagus* = Osteooph, *Phyllomedusa tomopterna* = Phyltomo, *Trachycephalus coriaceus* = Traccori, *Trachycephalus hadroceps* = Trachadr, *Trachycephalus resinifictrix* = Tracresi

Subsequently, we removed the effect of the cross factor event to show the temporal variability of the acoustic signals. Again, the first and second axes were strongly correlated with the peak frequency of the calls (r = 0.91 and r = 0.96, Pearson correlation). For both axes, low frequency calls lied on the negative side of the axis and high frequency calls on the positive one (Fig. 7). In this bi-dimensional space the acoustic community was structured along the temporal dimension (Fig. 7a). The first axis of the ordination analysis, with 60.1% of explained variance, showed a progression from t1 (negative side) to t4 (positive side), a progression toward mid-frequencies dominance. The levels t1 and t2 presented elongated ellipses, showing a dispersed range of frequency calls, with low and high-pitched sounds (Fig. 7a). This elongated shape was much less pronounced for levels t3 and t4, which was mainly characterized by calls in the mid-frequency range. The sounds that characterized, by their higher proportions, the explosive breeding event acoustics were the calls of *C. shudikarensis* and *T. coriaceus* (Fig. 7b). The calls of these anurans were in the middle range of the acoustic community, 3.4 kHz and 1.8 kHz for *C. shudikarensis* and *T. coriaceus* respectively.

**Fig. 8:**
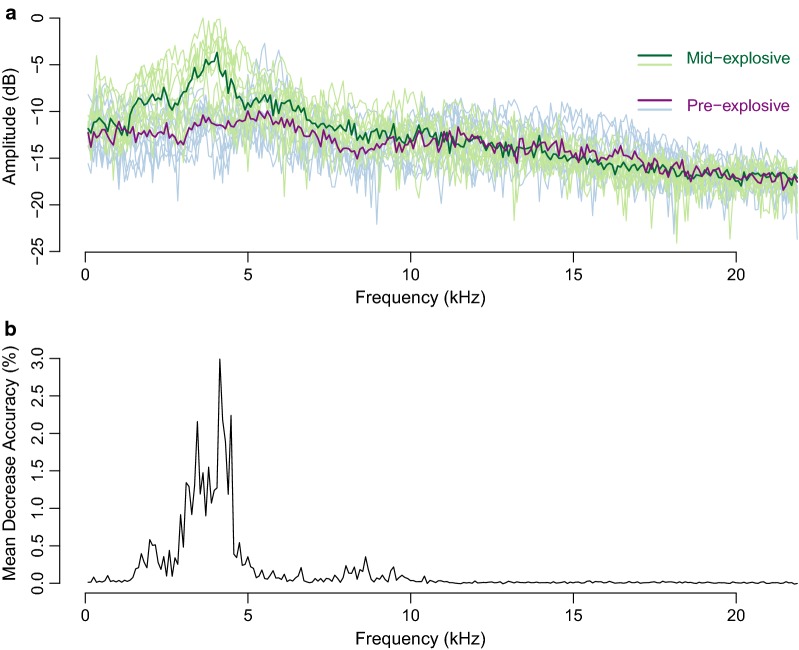
Comparison between spectral profiles of the acoustic communities before (pre) and during (mid) explosive breeding events. **a** In light colours, 10 random samples of each acoustic community, in dark purple (pre-explosive) and green (mid-explosive), the median spectrum of these communities. **b** Variable importance measure (mean decrease accuracy) from random forest classification used for discriminating the acoustics of explosive breeding events. Mid frequencies, between 3 and 4.4 kHz were the most important predictor variables

Further spectral analyses at the soundscape level supported the previous results obtained with isolated vocalisations. Using a statistical classifier we were able to classify explosive breeding recordings with high accuracy, using only their spectral profile (random forest, 89% out-of-the-bag accuracy). The feature importance analysis showed that mid frequencies, between 2 and 4.4 kHz, were clearly the most important predictor variables (Fig. 8).

**Fig. 9:**
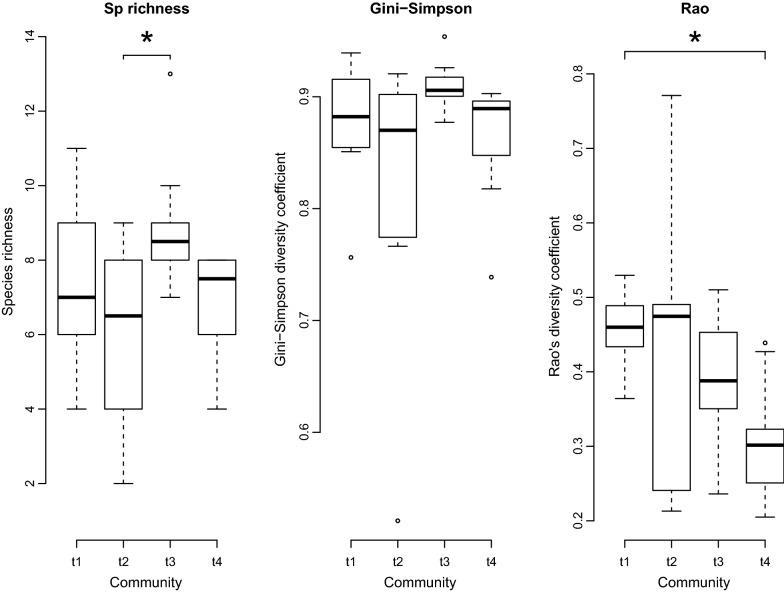
Diversity measures within each of the temporal community (t1, t2, t3 and t4) at each explosive breeding event (n = 10). Three diversity indices are compared: species richness, Gini–Simpson diversity and Rao’s diversity coefficient. While alpha diversity indices (species richness and Gini–Simpson index) showed similar values for pre-explosive (t1–t2) and explosive breeding communities (t4), Rao’s diversity index, which includes spectral distances between species, showed a significant diminution during explosive breeding events. Asterisk indicates significant differences between time periods

The temporal pattern observed using the species richness and the Gini–Simpson index was similar, with maximal values during the first hours of the explosive breeding event (period t3; Fig. 9). Differences in acoustic diversity among periods were statistically significant when measured as species richness (ANOVA, F_3,12_ = 5.86, p = 0.010) and marginally significant when measured by Gini–Simpson index (ANOVA, F_3,12_ = 3.21, p = 0.062). Post-hoc test revealed that the period t3 showed significantly higher species richness (2.8 ± 0.8) than the previous period t2 (Z = 3.51, p = 0.003), being others not statistically different. Rao’s diversity coefficient, which includes the acoustic dissimilarity matrix, also varied according to the time periods (ANOVA, F_3,12_ = 5.72 p = 0.011). This index was significantly higher at t1 than at t4 (0.15 ± 0.05; Z = 3.24, p = 0.007), indicating a progressive decrease in acoustic diversity as the explosive breeding community predominates (Fig. 9). No effect of the season nor its interaction with the periods of the event were identified in all cases (ANOVA, F_1,4_ < 4.48, p > 0.101), and hence the two explosive breeding events recorded per site, during each of the two rainy seasons, were equivalent in terms of acoustic diversity.

## Discussion

### Time series analysis

We found that environmental variables could predict the emergence of explosive breeding events, with rain as the most important predictor variable. While rain is abundant during the whole season, it is relevant to note that explosive breeding species respond to two specific patterns of rain: consistency during the previous 48 to 72 h and amount during previous 24 h. Our results are in agreement with those of Gottsberger and Gruber [27] who found that rainfall for the previous 24 h contributed the best, among other environmental variables, to explain the calling activity of the explosive breeding species. As we included more derived variables of the rain in our analyses, we complement previous results asserting that the consistency of the rain is also crucial. Having replicated this observation at several sites, we confirm that species participating in explosive breeding events are highly tuned to specific rainfall patterns. Recent studies have identified the photoperiod as an important predictor of anuran activity [20–23], but our statistical analyses showed no clear links between this factor and explosive breeding events. Our study site was very close to the equator (4°36′N), were the difference between maximum and minimum day length across the year is less than 32 min. Former studies on photoperiod were conducted at latitudes were the difference in day length are much more pronounced (at least 4.4 times stronger), which probably explains why this factor was so important.

This apparently high dependency of explosive breeders’ reproduction not only to the amount of precipitation but also to the timing of rain events raises the question of the vulnerability of explosive breeders to climate changes. While other factors such as programmed annual migration might be involved in triggering explosive breeding events, our study suggests that the two specific patterns of rain (i.e. consistency during the previous 48 to 72 h and amount during previous 24 h) are key parameters for the initiation of reproduction. With climate change increasing rainfall variability in tropical regions [53], reproductive events might be shifted or disrupted. Moreover, these species rely on very specific habitats (temporary reproductive ponds) for their reproduction that are very fragile and particularly vulnerable to climate changes [14]. Finally, the high number of individuals from several species at the time of reproduction might increase probability of intra and inter-species infection at the breeding ponds and therefore increase the sensitivity of these species to emerging infectious disease, in particular the fungus *Batrachochytrium dendrobatidis* [54]. These combined factors, may lead to significant shifts in the timing, distribution and composition of explosive breeding communities, which may desynchronize phenology and other biological responses throughout several trophic levels in the ecosystem [55].

### Community diversity analyses

In structurally similar habitats, located in the same region without obvious barriers, we expected to have homogeneous amphibian communities. Yet, our results highlight the variability of species composition in explosive breeding events. The ordination diagram showed differences in species composition both between ponds and for a given pond, between the two observed events. In other words, each explosive breeding event, while often sharing a common pool of species, had a unique combination of species. When controlling for the differences between explosive breeding communities, a clear temporal factor structured the acoustic community during explosive breeding events, showing pronounced differences between pre-explosive and explosive breeding communities. The main species characterising the explosive breeding event, *C. shudikarensis*, *T. coriaceus* and *C. cornuta*, were also found as predominant species in explosive breeding events in the Arataï river, more than 100 km away from our study site [27]. While other species are also present during these aggregations, these species seem particularly representative of the acoustic community.

It remains to explain the species turnover between events in space (ponds) and time (for each pond between the first and the second event). This turnover could be due to stochastic factors or related to multiple combined determinants, such as ecological and behavioural traits. As in many other sampling techniques in ecology, rare and elusive species are difficult to detect. It is also possible that the dense chorus of the louder species masked the vocalisations of more silent species, inducing detection errors and causing community variations in space and time.

### Acoustic diversity analyses

Regarding the acoustic environment of explosive breeding events, we found outlying levels of activity with a characteristic spectral signature. This signature stands out from the circadian acoustic environment and can be easily detected at long distance. Acoustic signatures convey information that could be exploited by conspecifics (or heterospecific) for general orientation within a landscape [56]. Fish and crustacean larvae [57], birds [58], and frogs [59, 60] are known to use sounds in the environment for spatial orientation. Indeed, acoustic cues might gain importance for anuran explosive breeding species since sounds may signal availability, in space and time, of short-lived breeding sites [60, 61].

Alpha diversity indices, measured with species richness and Gini–Simpson, showed temporal communities with similar values between pre-explosive (t1–t2) and the explosive breeding community (t4). The transitional community (t3) had higher values, probably because it had species from both communities, pre- and explosive breeding. More surprisingly, Rao’s diversity coefficient showed a significant diminution of the spectral diversity during explosive breeding events (t4). Even when the number of calling species was similar, we observed more frequency overlap in signals for the explosive breeding community than for the pre-explosive community.

Species belonging to a community may compete to access acoustic resources, that is to a free acoustic channel. It has been therefore hypothesized that species calling in a chorus should exhibit frequency dispersion. Formulated under the acoustic niche hypothesis, organisms would have evolved to occupy specific spectro-temporal ‘niches’, decreasing the risk of heterospecific mating and information masking [62]. Acoustic partitioning has been observed in multiple taxa, such as insects [63, 64], birds [65] and amphibians [66]. However, recent studies also presented limitations of such hypothesis, showing no significant spectral divergence in cricket assemblages [67] and more similarity in signal design that expected by chance for tropical forest birds [68]. Our results are in line with these last studies; contrary to our prediction, the species did not show frequency dispersion but frequency overlap.

Multiple hypotheses might explain this observation. First, the study ponds had similar habitat characteristics and hence similar acoustic properties that might have an effect on the features of anuran sounds. Following the acoustic adaptation hypothesis [69–71], the habitat might impose limits (e.g. signal attenuation and degradation) for sound propagation at high and low frequencies, resulting in an adaptation of explosive breeding species to produce sounds at mid frequencies, where they can maximize propagation. Indeed, for sounds produced at ground level, a window suitable for acoustic long range communication have been found at mid-frequencies (1–4 kHz) during experiments in an Amazonian rainforest in southern Venezuela [72]. Second, compared to prolonged breeders that show long periods of calling activity, explosive breeders share the acoustic space for very brief moments. As discussed by Wells [24], due to the short time window for exchanging vocal signals between individuals, males would rather compete physically and not acoustically. The selective pressures acting on the acoustic space of these species might be weaker, which could explain the high frequency overlap of the explosive breeding events. Finally, a convergence of signals, not only in time but also in frequency, could serve to better synchronise the sporadic emergence of multiple anuran species. A signal with common features across taxa would allow recruiting a larger number of individuals at precise location and time, aggregating organisms at densities that exceed the potential number of local predators. Indeed, studies on a variety of animals [73–75] and plants [76, 77] have shown that sporadic synchronous reproduction within a population significantly reduces levels of predation. However, to confirm a convergence on the signal, additional data should be included in the analysis, such as phylogenetic and functional traits. Moreover, sound propagation and playback experiments should be performed to shed light on the selective pressure driving widespread chorusing behaviour.

## Conclusions

In this study, we coupled biotic and abiotic variables, revealing community changes at multiple spatiotemporal scales and their tight link with the environment. Such data provides a baseline against which future changes can be measured, contributing to a better understanding and hopefully to a better management of such unique communities. Acoustic signatures could be used as a suitable way to monitor wildlife, not only at the individual or population level, but also at the community level, one of the main task of ecoacoustics [78]. A more widespread use of standardized methods combining passive acoustic recorders with a monitoring of key environmental parameters would become a comprehensible and cost-efficient framework to improve our knowledge and manage rich animal communities of tropical forests.

## Data Availability

The environmental audio recordings were deposited at the sound library of the Muséum national d’Histoire naturelle (https://sonotheque.mnhn.fr/).

## Abbreviations

ANOVA: analysis of variance
Ar: Arlesienne
Bl: Blanc
Ca: Caïman
DPCoA: double principle coordinate analysis
FFT: fast Fourier transform
GIS: geographic information system
IQR: inter quartile range
m a.s.l.: meters above sea level
Pa: Patawa
Pe: Petite
RMS: root mean square
SNR: signal to noise ratio
